# Supplementary material for: Physical Activity Barriers and Assets in Rural Appalachian Kentucky: A Mixed-Methods Study
Source: Int J Environ Res Public Health. 2021 Jul 19;18(14):7646. doi: 10.3390/ijerph18147646 (PMC8303275; doi:10.3390/ijerph18147646)
Supplement: Supplementary file 1 [file ijerph-18-07646-s001.zip › ijerph-1257555-supplementary.pdf]

Physical Activity Barriers and Assets in Rural Appalachian Kentucky: A Mixed Methods Study
Supplementary Materials

Instrument: Cohort Survey

We are conducting a research study to learn more about how your community and your habits may influence your dietary intake and physical activity patterns. We are asking you to take part in a 45-60-minute survey about your dietary habits, foods available in the home, physical activity, and some general information about yourself. You have previously agreed to participate in this survey by signing a consent form. It is funded by the Centers for Disease Control and Prevention. If at any time you do not wish to participate in this survey, you may stop. You may also skip questions that you are uncomfortable with answering. None of this information will be shared with your family members or friends. All information is kept private among study staff. You will be compensated for your time: all participants will receive a $25 gift card for each survey they participate in over the 3-year study period.

Questions About You

Name: ____

Address: ____ City: ___________________________

Zip Code: _________________________ Phone Number: ______________________________

Email: ____

If unable to reach you at the number listed above, is there another number we can contact to get in touch with you (a family member or friend)?

Option 1: ________________ Name: __________________

Option 2: ________________ Name: __________________

Height: _______ feet ______ inches **Weight**: __________ pounds

Please select one answer for the questions below.

What is your preferred language?

- English
- Spanish
- Other ___________

What is your gender?

- Male
- Female
- Transgender male to female
- Transgender female to male

What is your age in years? ________________

How long have you been a resident of Martin County?

- 1-3 years
- 4-7 years
- 8-10 years
- 10-20 years
- More than 20 years

Do you plan on moving to another county or out of the state in the next three years?

- Yes
- No
- Unsure

What is the highest grade of school you completed?

- 6th grade or below
- 7th-8th grade
- 9th-11th grade
- High school graduate or GED

- Some college
- College graduate

What is your race?

- American Indian
- Asian
- Black or African American
- Hispanic or Latino
- White
- Unknown
- Other

What is your annual **household** income?

- Less than $20,000
- $21,000-$39,999
- $40,000-$59,999
- $60,000-$79,999
- $80,000-$99,999
- More than $100,000

Do you have access to a vehicle to reach basic goods, services, or activities (i.e. travel to grocery store, post office, doctor's office)?

- Yes
- No

Do you own your own vehicle?

- Yes
- No

Are you currently pregnant or have you been pregnant in the last year?

- Yes
- No

Have you been diagnosed with a health concern in the last year that influenced your diet?

- Yes
- No

In the past month, did you or any member of your household receive SNAP (Supplemental Nutrition Assistance Program) benefits or food stamps?

- Yes
- No

In the past month, did you or any member of your household receive WIC (Women, Infants and Children) benefits?

- Yes
- No

In the past month, did you or anyone in your household receive Senior Farmers' Market Nutrition Program (SFMNP) vouchers?

- Yes
- No

Do you receive any other food assistance not listed above (i.e. food basket distribution programs)?

- Yes
- No

How often do you use Facebook?

- Never
- 1-2 times per month
- 1-2 times per week
- Daily

If you use Facebook, how do you access it?

- Smart phone/tablet
- Home computer or laptop
- Work computer or laptop
- Community computer or laptop

If you use Facebook, are you part of or would you use any groups?

- Yes
- No

Do you have a cell phone that receives text messages?

- Yes
- No

If your cell phone receives text messages, how comfortable are you texting?

- Very uncomfortable
- Uncomfortable
- Neutral
- Comfortable
- Very comfortable

<u>Attitudes</u>

The following questions ask about your attitudes toward fruits and vegetables.

Please indicate how much you like eating the following types of fruits and vegetables. Fruits and vegetables may be raw or cooked; fresh, canned, frozen, and dried or dehydrated; and may be whole, cut-up, mashed, or juiced.

1-I dislike them very much 2-I dislike them 3- I like them 4-I like them very much

	Dark green vegetables (broccoli, spinach, kale, collards, romaine)
	Starchy vegetables (potatoes, corn)
	Red and orange vegetables (carrots, bell peppers, tomatoes)
	Beans and peas (black, pinto, kidney, black-eyed peas, green peas)
	Other vegetables (cabbage, cucumbers, mushrooms, onions, celery)
	Citrus fruits (oranges, grapefruits, limes)
	Berries (strawberries, raspberries, blueberries)
	Melons (watermelon, cantaloupe, honeydew)
	Stone fruits (peaches, plums, apricots, cherries)
	Pome fruits (apples, pears)
	Tropical fruits (bananas, pineapples, mangoes)

Please indicate how much you like each of the following types of vegetables.

1-I dislike them very much 2-I dislike them 3- I like them 4-I like them very much

	Fresh vegetables (such as those you see immediately walking into a grocery store, or fresh from a garden)
	Frozen vegetables
	Canned vegetables

Please indicate how much you like each of the following types of fruits.

1-I dislike them very much 2-I dislike them 3- I like them 4-I like them very much

	Fresh fruits (such as those you see immediately walking into a grocery store, or fresh from a garden)
	Frozen fruits
	Canned fruits
	Dried fruits

Please indicate how much you agree or disagree with the following statements.

1-Strongly disagree 2- Disagree 3-Neither agree or disagree 4- Agree 5-Strongly agree

I would eat more vegetables…

	If my family ate more of them
	If the prices were cheaper
	If they were easier to prepare
	If they tasted better
	If more places close to me sold them
	If what I could buy were fresher at the store
	If I could keep them fresh longer

1-Strongly disagree 2- Disagree 3-Neither agree or disagree 4- Agree 5-Strongly agree

I would eat more fruit…

	If my family ate more of them
	If the prices were cheaper
	If they were easier to prepare
	If they tasted better
	If more places close to me sold them
	If what I could buy were fresher at the store
	If I could keep them fresh longer

Food Purchasing and Preparation

The next few questions are going to ask about purchasing habits and food preparation.
Are you the person who usually does the grocery shopping in your household?

- Yes
- No
- I split it with other household member(s)

Where do you get most of your groceries?

- Grocery store (IGA, Save-a-Lot, etc.)
- Super center (such as Wal-Mart)
- Discount stores (Dollar Store, Family Dollar, etc.)
- Drugstore or convenience store
- Other place

What is the main reason you shop there? Is it because of:

- Price
- Location
- Quality of products
- Variety of products
- Other

Are you aware of the farmers' market in your community?

- Yes
- No

Do you regularly shop at the farmers' market in your community?

- Yes
- No

Thinking back over the last week (seven days), did you purchase any: fast food, carry-out, delivery food, or prepared food from a deli? *Prepared foods from a deli include ready-to-eat foods from a grocery store deli department, such as rotisserie chicken, pizza, sandwiches, and salads from a salad bar.*

- Yes
- No

How many times in the last week (seven days), did you purchase fast food, carry-out, delivery food, or prepared food from a deli?

- None
- 1-3 times
- 4-6 times
- 7 or more times

Are you the person who usually prepares the meals in your household?

- Yes
- No

- I split it with other household member(s)

Which of the following statements best describes the amount of food eaten in your household in the last 30 days?

- Enough food to eat
- Sometimes enough to eat
- Often not enough to eat

Fruits and Vegetables

The following questions ask about how often you eat fruits and vegetables.

Think about all the fruits and vegetables that you ate last month. Include those that were: raw and cooked, eaten as snacks and at meals, eaten at home and away from home (restaurants, friends, take-out), and eaten alone and mixed with other foods.

Choose the **best** answer for each question. Mark only one response for each question.

1. Over the last month, how many times did you drink **100% juice** such as orange, apple, grape or grapefruit juice? Include juice you drank at all mealtimes and between meals.
DO NOT COUNT fruit drinks like Kool-Aid, Hi-C, lemonade, Sunny-D, or cranberry juice drink.

○ Never (Go to Question 2)	○ 1-3 times last month	○ 1-2 times per week	○ 3-4 times per week	○ 5-6 times per week	○ 1 time per day	○ 2 times per day	○ 3 times per day	○ 4 times per day	○ 5 or more times per day

1a. Each time you drank **100% juice**, how much did you usually drink?

○ Less than ¾ cup (less than 6 ounces)	○ ¾ cup to 1 ¼ cup (6 to 10 ounces)	○ 1 ¼ to 2 cups (10 to 16 ounces)	○ More than 2 cups (more than 16 ounces)

2. Over the last month, how many times did you eat **fruit**? Count any kind of fruit – fresh, canned, and frozen. **DO NOT COUNT** juices. Include fruit you ate at mealtimes and for snacks.

○ Never (Go to Question 3)	○ 1-3 times last month	○ 1-2 times per week	○ 3-4 times per week	○ 5-6 times per week	○ 1 time per day	○ 2 times per day	○ 3 times per day	○ 4 times per day	○ 5 or more times per day

2a. Each time you ate **fruit**, how much did you usually eat?

○ Less than 1 medium fruit (Less than ½ cup)	○ 1 medium fruit (About ½ cup)	○ 2 medium fruits (About 1 cup)	○ More than 2 medium fruits (More than 1 cup)

3. Over the last month, how often did you eat **lettuce salad (with or without other vegetables)**?

○ Never (Go to Question 4)	○ 1-3 times last month	○ 1-2 times per week	○ 3-4 times per week	○ 5-6 times per week	○ 1 time per day	○ 2 times per day	○ 3 times per day	○ 4 times per day	○ 5 or more times per day

3a. Each time you ate **lettuce salad**, how much did you usually eat?

○ About ½ cup	○ About 1 cup	○ About 2 cups	○ More than 2 cups

4. Over the last month, how often did you eat **French fries or fried potatoes**?

○ Never (Go to Question 5)	○ 1-3 times last month	○ 1-2 times per week	○ 3-4 times per week	○ 5-6 times per week	○ 1 time per day	○ 2 times per day	○ 3 times per day	○ 4 times per day	○ 5 or more times per day

4a. Each time you ate **French fries or fried potatoes**, how much did you usually eat?

○ Small order or less (About 1 cup or less)	○ Medium order (About 1 ½ cups)	○ Large order (About 2 cups)	○ Super Size order or more (About 3 cups or more)

5. Over the last month, how often did you eat **other white potatoes**? Count **baked, boiled** and **mashed potatoes, potato salad,** and **white potatoes that were not fried.**

○ Never (Go to Question 6)	○ 1-3 times last month	○ 1-2 times per week	○ 3-4 times per week	○ 5-6 times per week	○ 1 time per day	○ 2 times per day	○ 3 times per day	○ 4 times per day	○ 5 or more times per day

5a. Each time you had these **potatoes**, how much did you usually eat?

○ 1 small potato or less (About ½ cup or less)	○ 1 medium potato (½ to 1 cup)	○ 1 large potato (1 to 1 ½ cups)	○ 2 medium potatoes or more (1 ½ cups or more)

6. Over the last month, how often did you eat **cooked dry beans**? Count **baked beans, bean soup, refried beans, pork and beans,** and **other bean dishes.**

○ Never (Go to Question 7)	○ 1-3 times last month	○ 1-2 times per week	○ 3-4 times per week	○ 5-6 times per week	○ 1 time per day	○ 2 times per day	○ 3 times per day	○ 4 times per day	○ 5 or more times per day

6a. Each time you ate **these beans**, how much did you usually eat?

O Less than ½ cup	O ½ to 1 cup	O 1 to 1 ½ cups	O More than 1 ½ cups

7. Over the last month, how often did you eat **other vegetables?**
DO NOT COUNT: lettuce salads, white potatoes, cooked dried beans, vegetables in mixtures, such as on sandwiches, in omelets, casseroles, stews, stir-fry, soups, etc. or rice
COUNT: all other vegetables – raw, cooked, canned, and frozen – examples include cabbage, carrots, broccoli, cauliflower, cucumber, tomatoes, etc.

O Never (Go to Question 8)	O 1-3 times last month	O 1-2 times per week	O 3-4 times per week	O 5-6 times per week	O 1 time per day	O 2 times per day	O 3 times per day	O 4 times per day	O 5 or more times per day

7a. Each time you ate **other vegetables**, how much did you usually eat?

O Less than ½ cup	O ½ to 1 cup	O 1 to 2 cups	O More than 2 cups

8. Over the last month, how often did you eat **tomato sauce**? Include tomato sauce on pasta or macaroni, rice, pizza, and other dishes.

O Never (Go to Question 9)	O 1-3 times last month	O 1-2 times per week	O 3-4 times per week	O 5-6 times per week	O 1 time per day	O 2 times per day	O 3 times per day	O 4 times per day	O 5 or more times per day

8a. Each time you ate **tomato sauce**, how much did you usually eat?

O Less than ¼ cup	O About ½ cup	O About 1 cup	O More than 1 cup

9. Over the last month, how often did you eat **vegetable soups**? Include tomato soup, beef with vegetable soup, or other soups made with vegetables.

O Never (Go to Question 10)	O 1-3 times last month	O 1-2 times per week	O 3-4 times per week	O 5-6 times per week	O 1 time per day	O 2 times per day	O 3 times per day	O 4 times per day	O 5 or more times per day

9a. Each time you ate **vegetable soup**, how much did you usually eat?

O Less than 1 cup	O 1 to 2 cups	O 2 to 3 cups	O More than 3 cups

10. Over the last month, how often did you eat **mixtures that included vegetables**? Count foods such as sandwiches, casseroles, stews, omelets, and tacos.

Never	1-3 times last month	1-2 times per week	3-4 times per week	5-6 times per week	1 time per day	2 times per day	3 times per day	4 times per day	5 or more times per day

In a usual week, do you feel that you eat as many fruits and vegetables as you need?

a. Yes, I definitely do
b. Yes, I think so
c. I'm not sure
d. No, I don't think so
e. No, I definitely do not

Beverage Questionnaire

In the past month, please indicate your response for each beverage type by marking an "X" in the box for "how often" and "how much each time"

1. Indicate how often you drank the following beverages, for example, you drank 5 glasses of water per week, therefore mark 4-6 times per week
2. Indicate the approximate amount of beverage you drank each time, for example, you drank 1 cup of water 2 times per day, therefore mark 1 cup under "how much each time"

	HOW OFTEN (MARK ONE)							HOW MUCH EACH TIME (MARK ONE)				
**Type of beverage**	Never (skip to next)	1 time per week	2-3 times per week	4-6 times per week	1 time daily	2 times per day	3+ times per day	Less than 6 fl. ounces (¾ cup)	8 fl. ounces (1 cup)	12 fl. ounces (1 ½ cups)	16 fl. ounces (2 cups)	20 fl. ounces or more (2 ½ cups)
Bottled water												
Tap water												
100% fruit juice (apple, orange, grape)												
Sweetened juice (lemonade, Sunny-D, Kool-Aid, Hi-C)												
100% vegetable juice (tomato, V8)												
Whole milk												
Reduced fat milk (2%)												
Low-fat/fat-free milk (1%, skim, buttermilk, soymilk)												
Milk alternatives (almond, soy, coconut milk)												
Soft drinks, regular												

Diet soft drinks												
Sweet tea												
Unsweet tea												
	HOW OFTEN (MARK ONE)							HOW MUCH EACH TIME (MARK ONE)				
**Type of beverage**	Never (skip to next)	1 time weekly	2-3 times weekly	4-6 times weekly	1 time daily	2 times daily	3+ times daily	Less than 6 fl. ounces (¾ cup)	8 fl. ounces (1 cup)	12 fl. ounces (1 ½ cups)	16 fl. ounces (2 cups)	20 fl. ounces or more (2 ½ cups)
Coffee, with cream and/or sugar												
Coffee, without cream and/or sugar												
Beer, ales, wine coolers												
Hard liquor (whiskey, rum, tequila, etc.)												
Mixed alcoholic beverages (daiquiris, margaritas, etc.)												
Wine (red or white)												
Meal replacement shakes/protein drinks (Slimfast, Spark, Shakeology)												
Artificially sweetened drinks (Crystal Light)												
Energy drinks (Red Bull, Rockstar, Monster, etc.)												

Water: The next few questions are going to ask about water, specifically, in your household.
What is the main source of drinking-water for your household?

- Tap water
- Bottled water
- Rainwater
- Other

What is the main source of your home tap water?

- City
- Well
- Spring

- Other

Do you feel that your home tap drinking water is safe to drink?

- Yes
- No

Do you use your home tap water for cooking or other purposes, such as laundry and hand-washing?

- Yes
- No

What do you feel the quality of water is where you live?
- Poor, and not improving
- Poor, but improving
- Good
- Good, and improving
- Excellent

Physical Activity: The following questions will ask you about your current level of physical activity.

In a typical week, do you do any **vigorous-intensity** sports, fitness, or recreational activities that cause **large increases** in breathing or heart rate for at least **10 minutes continuously?** Examples include: running, hiking, shoveling, or playing basketball.
- Yes
- No

If you answered "Yes" to the previous question, on how many days per week did you participate in these activities?
- 1-2 days
- 3-4 days
- 5-6 days
- 7 days

In a typical week, do you do any **moderate-intensity** sports, fitness, or recreational activities that cause **small increases** in breathing or heart rate for at least **10 minutes continuously?** Examples include: brisk walking, bicycling, gardening, or heavy cleaning.
- Yes
- No

If you answered "Yes" to the previous question, on how many days per week did you participate in these activities?
- 1-2 days
- 3-4 days
- 5-6 days
- 7 days

Thinking about the past year, how much do the following items prevent you from participating in at least 30 minutes of activity per day?

1-Not at all 2-Slightly 3-Quite a bit 4-A great deal

	Lack of time
	Lack of energy or motivation
	Lack of space
	Access to reliable childcare
	Access to facilities or space to be active
	Access to proper clothing or shoes for activity
	Access to safe places to walk
	Cost
	Weather
	Self-conscious
	Health condition (such as asthma, COPD, or arthritis)
	Injury (such as a broken bone, recovery from surgery)
	Lack of self-discipline

How much do you agree or disagree with the following statements that may influence your activity levels?

1-Strongly disagree 2-Disagree 3-Neither agree or disagree 4-Agree 5-Strongly agree

	I do not have the time for physical activities

	I do not have the energy for physical activities
	I feel sick while exercising
	I find physical activities to be difficult and tiring
	Physical activity makes me feel good about myself
	I have health conditions that prevent me from being physically active

1-Strongly disagree 2-Disagree 3-Neither agree or disagree 4-Agree 5-Strongly agree

	I have concerns about injury when exercising
	I experience discomfort during or after exercising
	I fear for my safety when exercising
	I look funny or feel ashamed when doing physical activities
	I feel physical activities improve my health
	I find physical activity makes me sore and uncomfortable

1-Strongly disagree 2-Disagree 3-Neither agree or disagree 4-Agree 5-Strongly agree

	I don't have anyone to take care of my children or family members
	I have friends who are physically active
	My friends will be active with me
	I have family members who are physically active
	My family members will be active with me
	I don't have free time to do physical activities because of my work schedule
	There are no places or facilities to walk or do physical activities near where I live
	Places or facilities where I can walk and be active are easy to get to
	I do not have transportation to places or facilities where I can walk and be active
	The activities I want to do cost too much
	I don't know how to use equipment while doing physical activities
	I don't have the right clothes or shoes for walking or physical activities I want to do
	The weather prevents me from doing physical activities
	I find there are no benches or places to rest while being active

Community
The next set of questions ask about your community. Community can include people in your town, neighborhood, county, faith congregation, or local organization.
How much do you agree or disagree with each of the following statements?
1-Strongly disagree 2-Disagree 3-Neither agree or disagree 4-Agree 5-Strongly agree

	Adult obesity is a problem in my community
	Not having access to places to be active and to do things like walk, bike, and play is a problem in my community.
	Not getting enough healthy foods and drinks is a problem in my community.
	It is important for my community to be involved in helping solve the issue of not getting enough healthy foods and drinks.
	My community should do more to make it easier for adults to get healthy foods.
	My community should do more to make it easier for adults to be active and do things like walk, bike, and play.
	I would like employers in my community to make healthy foods available when foods are served at meetings or sold in cafeterias or vending machines

How much do you agree or disagree with each of the following statements?
1-Strongly disagree 2-Disagree 3-Neither agree or disagree 4-Agree 5-Strongly agree

	I would like child care and education programs in my community to follow healthy food guidelines
	I would like my community to work with farmers to make healthy food more available and accessible
	I would like organizations in my community – such as faith-based organizations and schools – to make healthy foods available when food is being served
	I would like vending machines to offer more healthy foods
	I would like healthy foods to be more available in my community

Are you aware of any program(s) in your local community that are encouraging people to eat healthy foods, drink healthy beverages, be more physically active, or work together to make these things easier to do? **Please select one.**

	Yes
	No
	I don't know

How important is it to you that your community has the following?
1-Not at all important 2-Slightly important 3-Moderately important 4-Very important 5-Extremely important

	Sidewalks or other types of walking paths
	Dedicated bike lanes
	Parks and recreation area s

Awareness
In the past 30 days have you seen, heard, or read any TV, radio, newspaper, or online advertising about any of the following in your county? Select all that apply.

	Encouraging individuals to eat healthy food or to drink healthy drinks
	Encouraging individuals to do physical activities
	Increasing local healthy food and drink options
	Increasing local physical activity options
	Making it easier in communities to walk, bike, and play
	Getting more physical education and physical activity for children in schools
	Making it easier for children to drink more water throughout the day

In the past 30 days, how often have you seen or heard any messages that encourage individuals in your county to eat healthy foods, drink healthy beverages, and/or be physically active? Please select one.

	1. Not at all
	2. Once in the past 30 days
	3. A few times in the past 30 days
	4. Many times in the past 30 days
	5. Daily or almost daily in the past 30 days
	6. Don’t know

If answered 2-5 above:
Thinking of the messages that you saw or heard that encouraged individuals to eat healthy foods, drink healthy beverages, and/or be physically active, where did you see or hear them? Select all that apply.

	Newspapers or magazines
	Billboards or posters
	TV or Radio
	Websites
	Social media (Facebook, Twitter, Instagram, etc.)
	Promotion at work
	Promotion at sports or community event
	Somewhere else not listed
	I do not recall seeing messages about these topics

Instrument: Focus Group Moderator Guide

Participants will be asked the following questions to gain an understanding of knowledge of resources available for making healthy choices in the community, the need for additional resources to promote healthy choices, and the barriers and facilitating factors to support healthy eating habits and physical activity in their community.

Introduction Script:
Good morning / afternoon. Thank you for taking the time to be here today. My name is ____________ and I will be leading this focus group. The notetakers and I are from the University of Kentucky Cooperative Extension Service. Your participation is greatly appreciated, and we hope to have a good discussion this morning / afternoon. Please silence your cell phones so we may have fewer distractions during our time together.
My role is to ask questions and keep the conversation moving. We want to create a safe place for everyone to share their opinion, so please be respectful and let people finish their thoughts before responding. We encourage you to share your perspective, even if it is different from others. There are no right or wrong answers to the questions asked, only different points of view. You are free to participate as much or as little as you feel comfortable. We have one note taker sitting in the back of the room and will also be tape recording this focus group session.
We are here today to talk about healthy eating and active living. We would like to identify or better understand any facilitating factors and barriers to making healthy choices in your community. The responses you share today will help us identify needs for future programs in the community.
Let's begin by going around the room. Please introduce yourself by first name only and state your role i.e. grocery store manager, local farmer, coalition member, community resident etc.

End Script:
Thank you very much for your time. It is important for us to understand the available resources for healthy choices within a community, as well as what needs to be developed. Your answers are important and useful to us. We appreciate your time and thank you again for attending and sharing your opinions.

Questions:
"The first set of questions we are going to ask are related to healthy food choices. These questions will be related to resources in your community, the need for additional resources, and any challenges you may have to make healthy choices in your community."

1. "How easy it is to get healthy foods in your community? Where are the places you can purchase food in your community?"
2. "How easy or difficult is it for you to purchase healthy food on a regular basis?"
3. "What do you think are some of the community's greatest assets when it comes to eating healthy food?"
4. "What resources are available for people to eat healthy in your community?"
5. "Do you think your community is designed to promote healthy eating choices? Why or why not?"
6. "Is purchasing produce something that is important to you? Why or why not? How easy is it for you to find the produce you want?"
7. "In your opinion, what factors in your community contribute to a lack of healthy eating?"
8. "What changes could be made in your community to help people make healthy/healthier choices?"
9. "What resources are currently available in your community that can help improve access to produce?"
10. "What types of food resources do you think would be helpful to have in your community to allow people to purchase healthy food?"
11. "What would be some ways to motivate or encourage people in your community to eat healthy foods?"
12. "If you could do anything to make it easier to make healthy choices in your community, what would you do?"
13. "If there was greater availability of healthy food, what other strategies or resources would be needed to help community members purchase and consume these items?"

14. “Who are the key people in the community when it comes to providing healthy foods?”

“We are now going to transition to a few questions related to physical activity. As with those we just went through, these questions will be related to resources in your community, the need for additional resources, and any challenges to being physically active in your community.”

15. “How easy or difficult is it for you to be physically active in your community on a regular basis?”
16. “Do you feel like your community encourages physical activity? Why or why not?”
17. “What resources are available in your community that allow you to be physically active?
18. “Do you regularly use resources available for activity in your community? Why or why not?”
19. “How safe do you feel utilizing resources available in your community?”
20. “What changes would you make to your community to encourage more physical activity?”
21. “What are some of the common everyday destinations in your community? What routes do you use to reach these destinations?”
22. “Of the destinations and routes you shared, are there spaces that feel more safe than others? Why or why not?”
23. “When thinking about ways to make it easier to engage in some form of physical activity, what would you like to have access to, or see more of in your community for yourself and/or your family?”

“Thank you for your thoughtful responses so far. We want to end our time together by asking a couple of summary questions.”

24. “Of all of the things we have talked about today, what is the most important to you?”
25. “Is there anything we should have talked about, but didn’t?”
